# Supplementary material for: Transcriptional bursting is intrinsically caused by interplay between RNA polymerases on DNA
Source: Nat Commun. 2016 Dec 7;7:13788. doi: 10.1038/ncomms13788 (PMC5151093; doi:10.1038/ncomms13788)
Supplement: Supplementary Information — Supplementary Figures 1-8, Supplementary Table 1 and Supplementary References [file ncomms13788-s1.pdf]

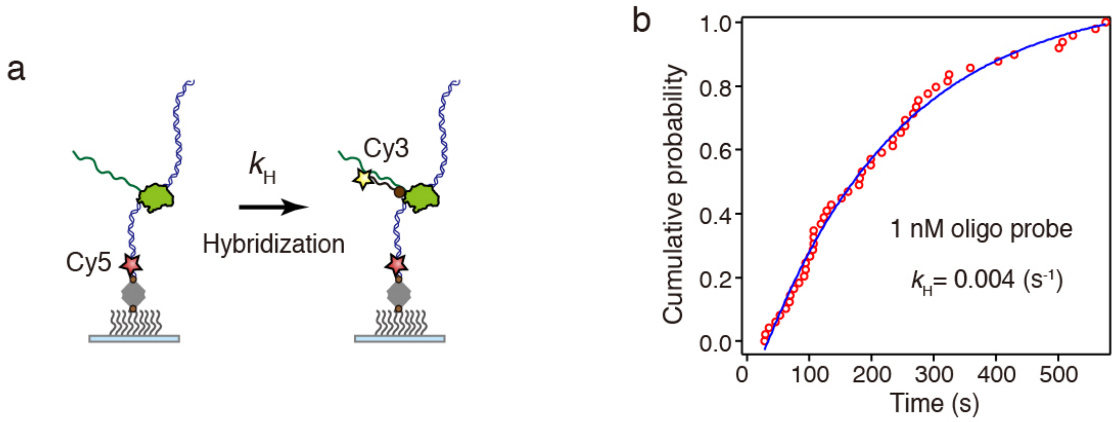

Supplementary Figure 1. Hybridization rate of the oligo probe. (**a**) Measurement of the oligo probe hybridization rate. After an *in vitro* transcription was performed at 10 nM RNAP concentration and the nucleotide concentration of 10[*NTP*] (100 μM GTP & UTP, 50 μM ATP, 25 μM CTP) for 10 min at 25℃, the hybridization time on the nascent mRNA was measured by adding 1 nM oligo probe. (**b**) Cumulative frequency of hybridization time of oligo probe, giving the hybridization rate, $k_H = 4 \times 10^6$ M^{-1} s^{-1} (0.004 s^{-1} at 1 nM oligo probe), which is comparable with the reported value, $k_H = 6 \times 10^6$ M^{-1} s^{-1} [1]. $N = 50$. Data were obtained from two independent successfully reproduced experiments.

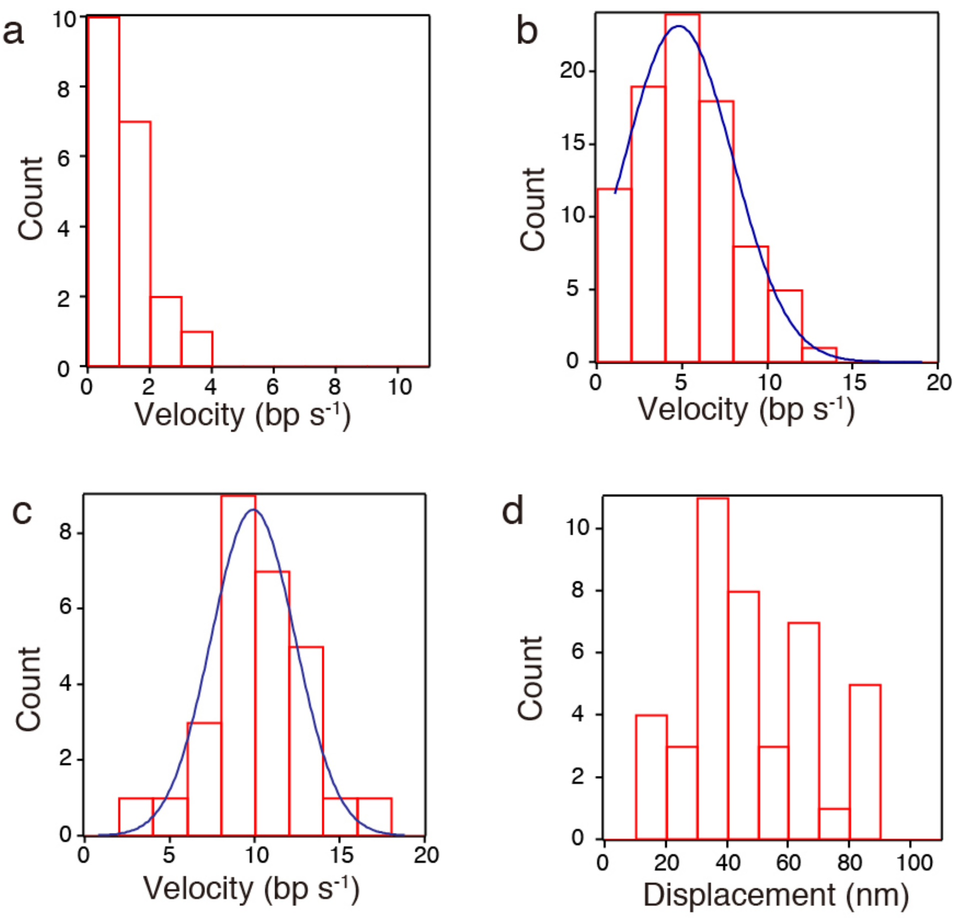

Supplementary Figure 2. Elongation velocity in the Qdot tracking experiment. (**a-c**) Distributions of velocity at 1[*NTP*] (10 μM GTP & UTP, 5 μM ATP, 2.5 μM CTP) (*N* = 20) in **a**, 10[*NTP*] (100 μM GTP & UTP, 50 μM ATP, 25 μM CTP) (*N* = 87) in **b**, and 100[*NTP*] (*N* = 38) in **c**. Gaussian fits (blue line) give a peak of 4.7 ± 4.5 bp s^{-1} in **b** and 10 ± 3.5 bp s^{-1} in **c** (± s.d.). (**d**) Distribution of displacement during elongation with a T7A1 promoter (**Fig. 1b**) at 100[*NTP*]. *N* = 42. All data sets were obtained from more than three independent successfully reproduced experiments.

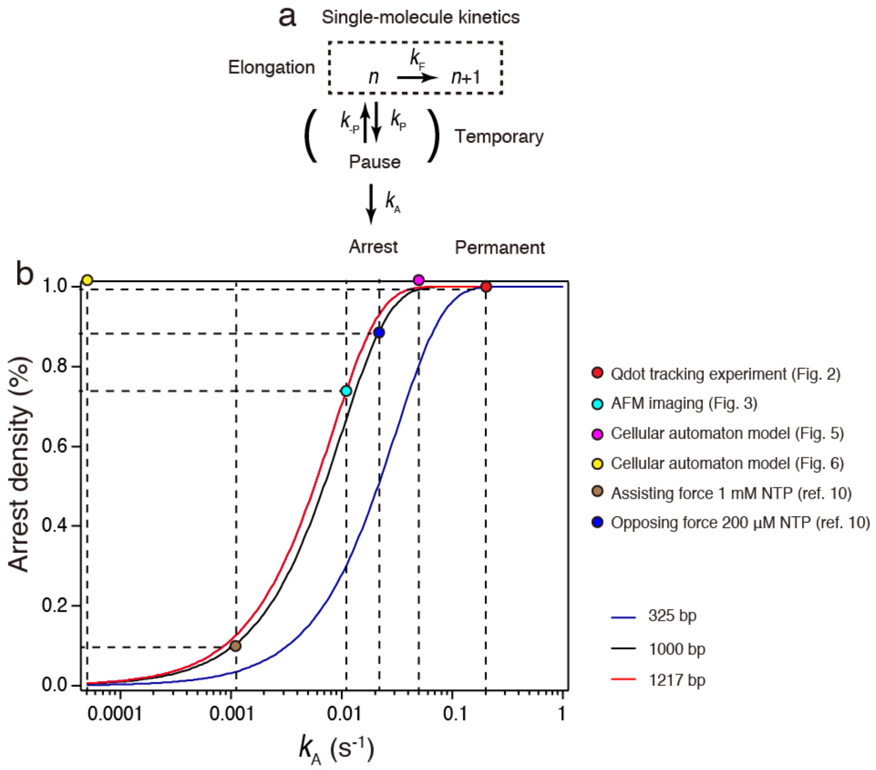

Supplementary Figure 3. Estimation of arrest rate (k_A). (**a**) The processive movement of RNAP is not continuous but is stochastically interrupted by transcriptional pauses[2-4]. This pause is temporary, but the dwell time can be increased by backtracking, and when coupled with fraying of the 3’ end of nascent RNA from the active site of RNAP, leads permanent halt (arrest)[5-7]. This off-pathway from transcription elongation was approximated by the simple kinetic scheme in which the relevant parameters were measured by *in vitro* single-molecule measurements for a single RNAP molecule[8-10]. (**b**) Assuming the simple scheme, where RNAP is arrested with a constant rate (k_A) at each base pair, arrest density can be described by the following equation.

$$\boldsymbol{arrest\ density}\ (\%) = \mathbf{1} - \left(\mathbf{1} - \frac{\boldsymbol{k}_{\mathbf{A}}}{\boldsymbol{k}_{\mathbf{F}} + \boldsymbol{k}_{\mathbf{A}}}\right)^{\boldsymbol{L}}$$

Here, arrest density is the probability that a single RNAP molecule is arrested during elongation of L (bp). When $\boldsymbol{k}_F = 10\ s^{-1}$ (**Supplementary Fig. 2**) and $\boldsymbol{k}_F >> \boldsymbol{k}_A$,

$$\boldsymbol{arrest\ density}\ (\%) = \mathbf{1} - \left(\mathbf{1} - \frac{\boldsymbol{k}_{\mathbf{A}}}{\mathbf{10}}\right)^{\boldsymbol{L}}$$

Based on this definition, we estimated k_A in our experiments and the previous report[10] (**Supplementary Table. 1**).

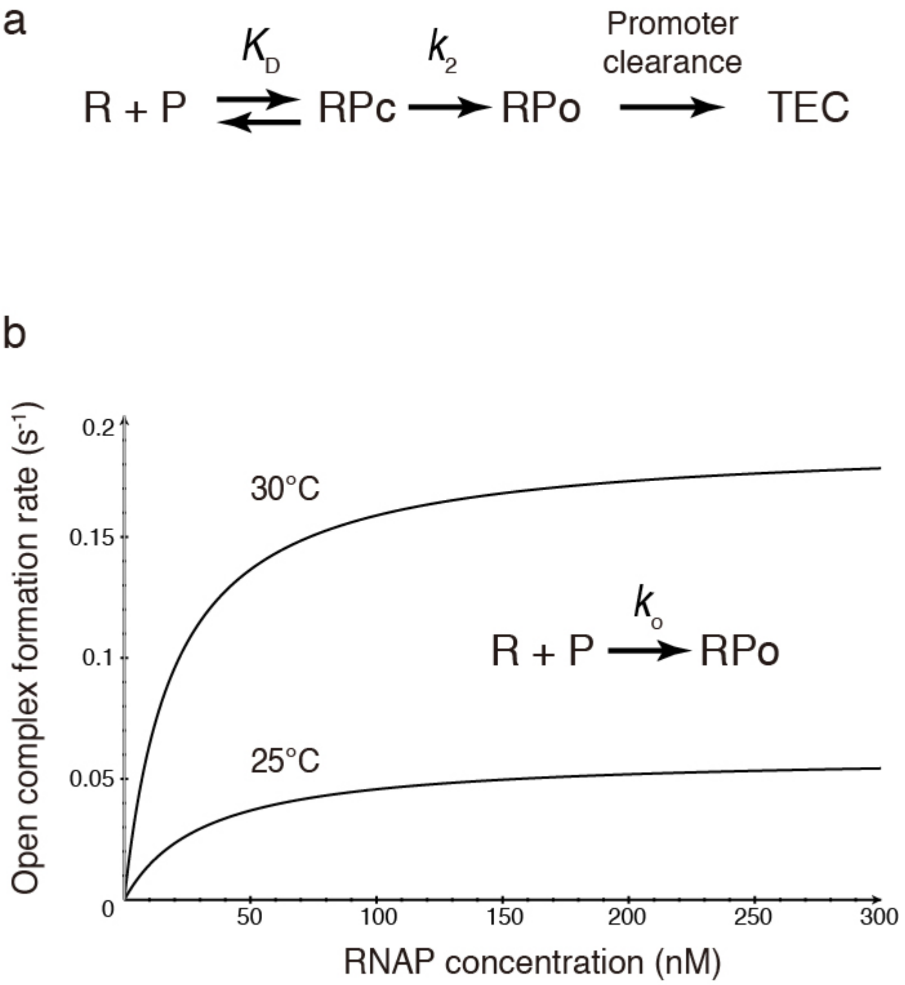

Supplementary Figure 4. Estimation of transcription initiation rate (k_I). (**a**) Kinetic scheme for transcription initiation[11]. (**b**) We calculated the open complex formation rate by the following equation[12].

$$\boldsymbol{k_o} = \frac{\boldsymbol{k_2[RNAP]}}{\boldsymbol{[RNAP] + K_D}}$$

Here, we obtained $K_D = 31.3 \times 10^{-9}$ M and 19.6×10^{-9} M, and $k_2 = 0.06$ s^{-1} and 0.19 s^{-1} at 25℃ and 30℃, respectively from the reference[13]. Based on our experimental data that mRNA production rate depends on the open complex formation rate, we assume that the open complex formation rate is the rate-limiting step of transcription initiation at least in our experimental condition (25℃ or 30℃, and 100[*NTP*]). Therefore, we defined the open complex formation rate as the transcription initiation rate.

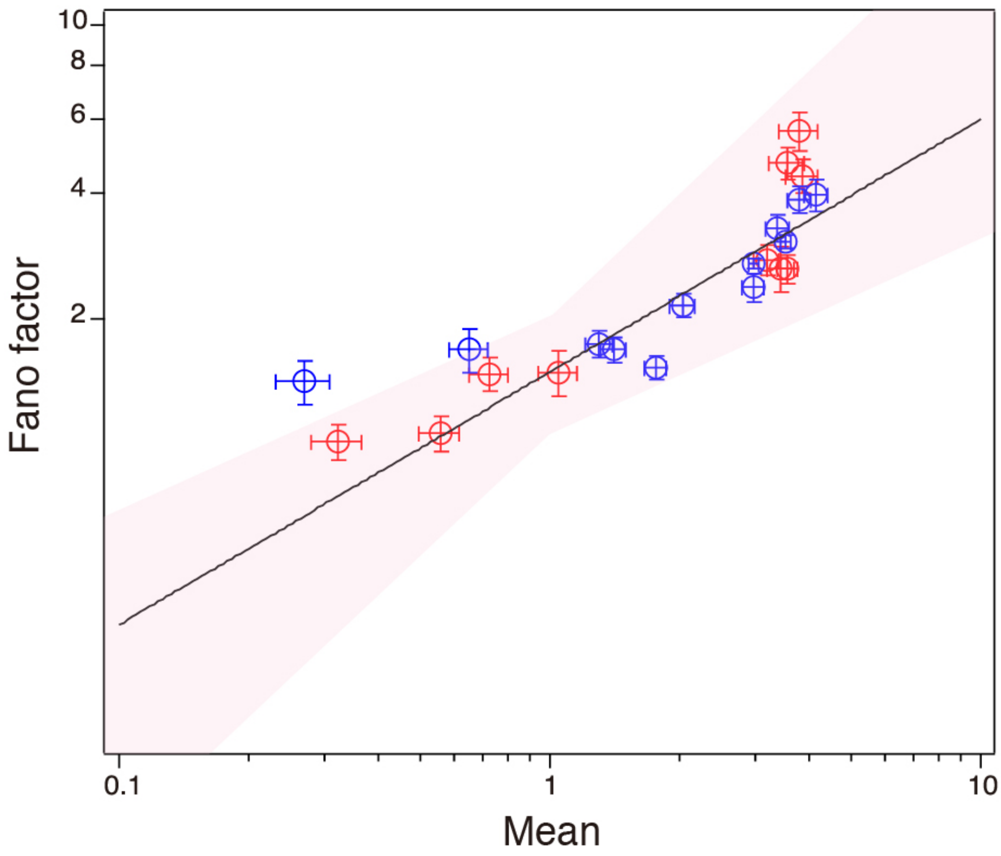

Supplementary Figure 5. Log-log plot of mean and the Fano factor (Fig. 4l). The red plot (experimental data) was described by $b = k\langle m\rangle^{\alpha}$ with k =1.6 ± 0.5 and α = 0.71 ± 0.25 (± s.d.). The blue plot indicates the simulated data. The parameter error is represented by the red region.

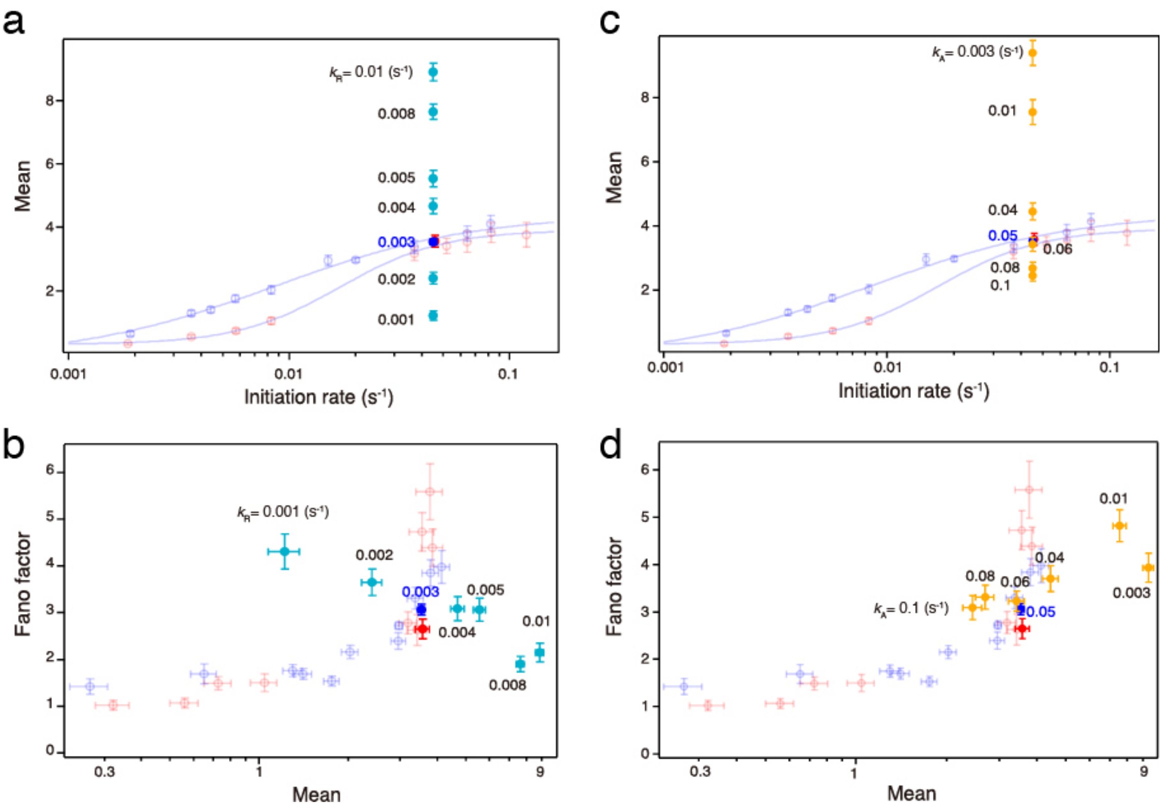

Supplementary Figure 6. Modulation of rescue rate (k_R) and arrest rate (k_A) in the cellular automaton model. (**a,b**) The effect on the simulated mean and Fano factor by modulation of k_R in the cellular automaton model (turquoise circle). The simulated data best reproduced the mean and Fano factor of the experimental distribution (k_I = 0.045 s^{-1}, red circle) by k_R = 0.003 s^{-1} (blue circle). Error bars indicate the bootstrapped standard deviation. Mean and Fano factor were calculated by the simulated histograms composed of 250 data sets. The plots were overlaid on **Figure 4k,l**. (**c,d**) Effect on the simulated mean and Fano factor by modulation of k_A in the cellular automaton model (orange circle). The simulated data best reproduced the mean and Fano factor of the experimental distribution (k_I = 0.045 s^{-1}, red circle) by k_A = 0.05 s^{-1} (blue circle). Error bars indicate the bootstrapped standard deviation. Mean and Fano factor were calculated by the simulated histograms composed of 250 data sets. The plots were overlaid on **Figure 4k,l**.

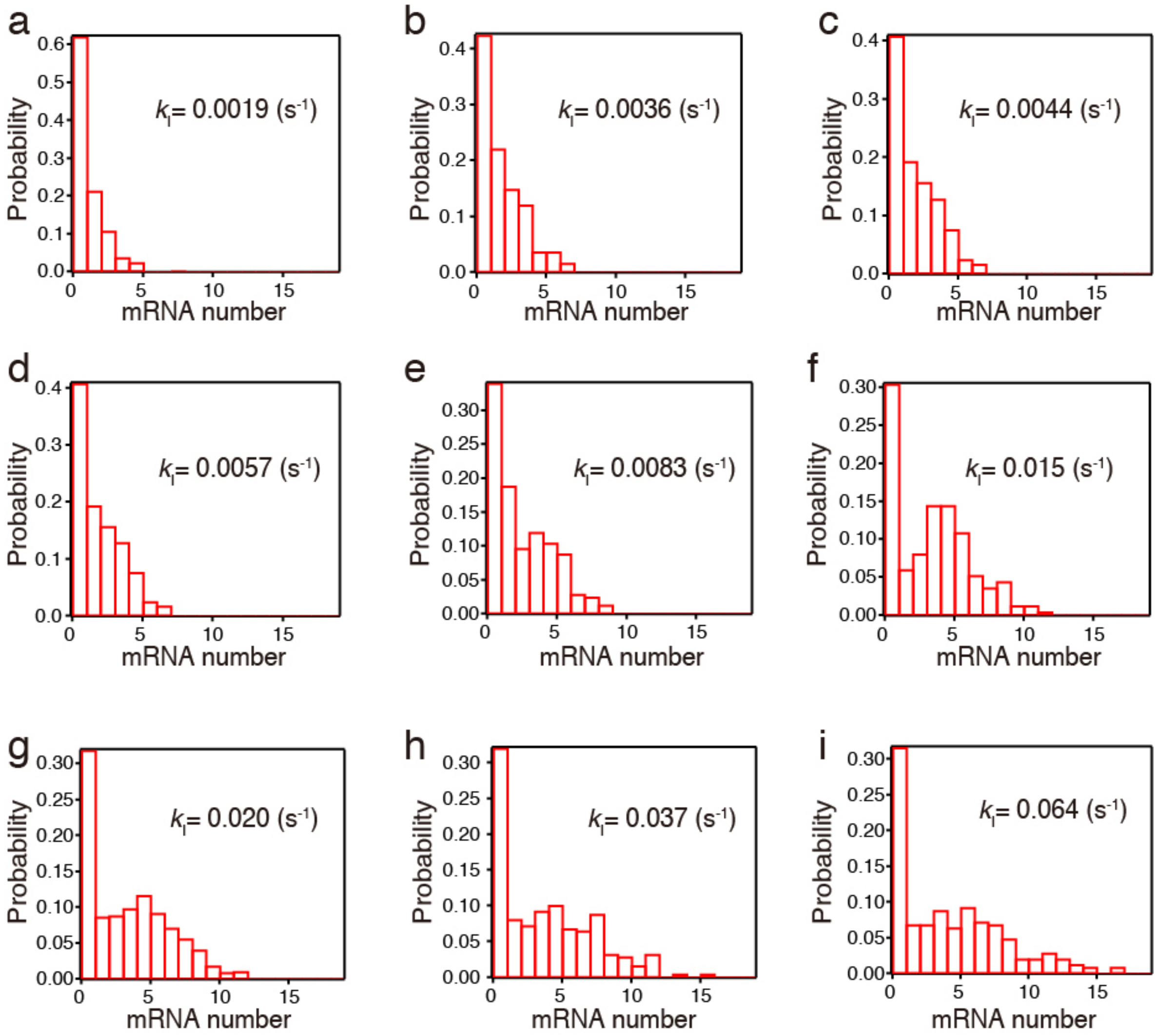

Supplementary Figure 7. Simulated mRNA number distributions. (**a-i**) mRNA number distributions obtained by the cellular automaton model with the different transcription initiation rates. $N = 250$ except for **g** ($N = 1250$ in **g**).

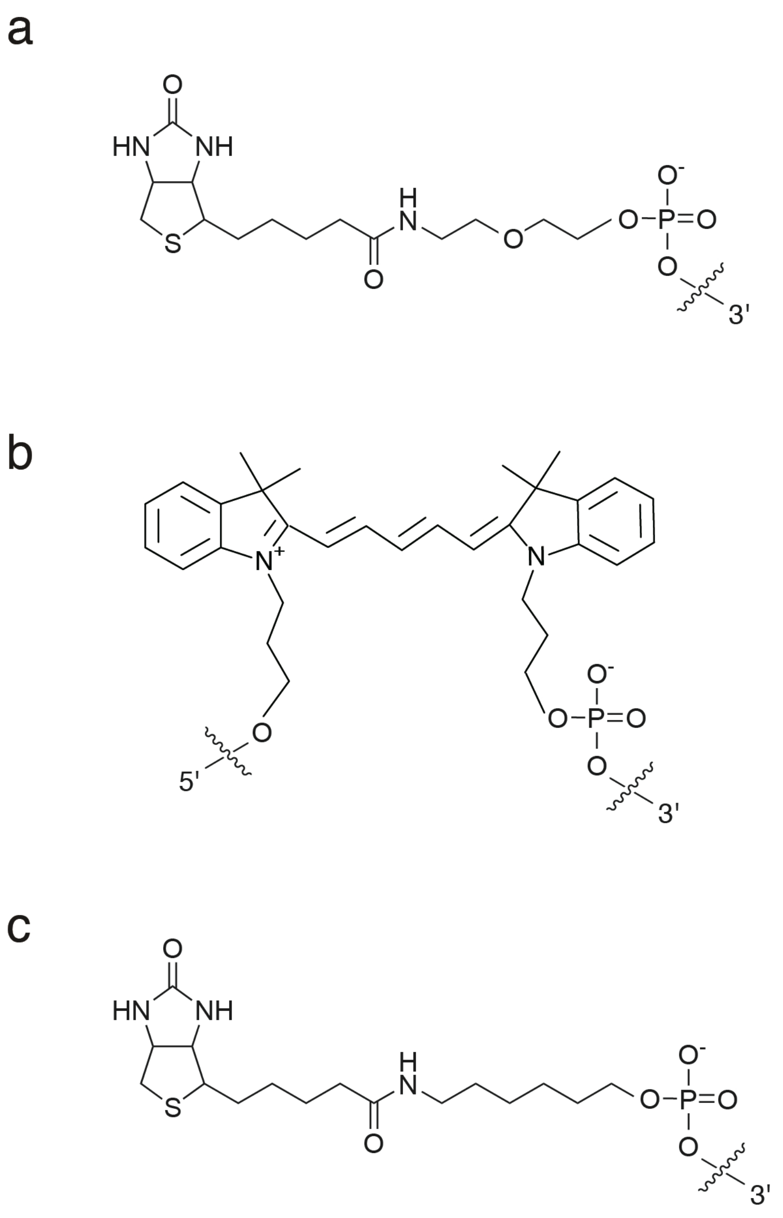

Supplementary Figure 8. The structures of the primer modifications. (**a-b**) The structures of the modifications in biotin (**a**) and Cy5 (**b**), in the primer, 5'-/5Bio/G/Cy5/CCGGATAAAACTTGTGC-3' (synthesized by Integrated DNA technologies, IDT, Coralville, IA). (**c**) The structure of the biotin modification in the primers, 5'-/5Bio/CCACAACGGTTTCCCTCTAG-3' and 5'-/5Bio/GCCGGATAAAACTTGTGC-3' (synthesized by Hokkaido System Science Co., Ltd, Japan).

Supplementary Table 1. **Estimation of arrest rate (k_A) based on the scheme (Supplementary Fig. 3a)**

Qdot tracking experiment (**Fig. 2**)	~0.2 s^{-1}
AFM imaging (**Fig. 3**)	0.011 s^{-1}
Cellular automaton model (**Fig. 5**)	0.05 s^{-1}
Cellular automaton model (**Fig. 6**)	0.00005 s^{-1}
Assisting force 1 mM NTP (ref. 10)	0.0012 s^{-1}
Opposing force 200 μM NTP (ref. 10)	0.02 s^{-1}

Supplementary References

1. Zhang, Z., Revyakin, A., Grimm, J.B., Lavis, L.D. & Tjian, R. Single-molecule tracking of the transcription cycle by sub-second RNA detection. *Elife* **3**, e01775 (2014).

2. Herbert, K.M. et al. Sequence-resolved detection of pausing by single RNA polymerase molecules. *Cell* **125**, 1083-1094 (2006).

3. Larson, M.H. et al. A pause sequence enriched at translation start sites drives transcription dynamics in vivo. *Science* **344**, 1042-1047 (2014).

4. Vvedenskaya, I.O. et al. Interactions between RNA polymerase and the "core recognition element" counteract pausing. *Science* **344**, 1285-1289 (2014).

5. Komissarova, N. & Kashlev, M. Transcriptional arrest: Escherichia coli RNA polymerase translocates backward, leaving the 3' end of the RNA intact and extruded. *Proc Natl Acad Sci U S A* **94**, 1755-1760 (1997).

6. Cheung, A.C. & Cramer, P. Structural basis of RNA polymerase II backtracking, arrest and reactivation. *Nature* **471**, 249-253 (2011).

7. Weixlbaumer, A., Leon, K., Landick, R. & Darst, S.A. Structural basis of transcriptional pausing in bacteria. *Cell* **152**, 431-441 (2013).

8. Wang, M.D. et al. Force and velocity measured for single molecules of RNA polymerase. *Science* **282**, 902-907 (1998).

9. Davenport, R.J., Wuite, G.J., Landick, R. & Bustamante, C. Single-molecule study of transcriptional pausing and arrest by E. coli RNA polymerase. *Science* **287**, 2497-2500 (2000).

10. Forde, N.R., Izhaky, D., Woodcock, G.R., Wuite, G.J. & Bustamante, C. Using mechanical force to probe the mechanism of pausing and arrest during continuous elongation by Escherichia coli RNA polymerase. *Proc Natl Acad Sci U S A* **99**, 11682-11687 (2002).

11. McClure, W.R. Mechanism and control of transcription initiation in prokaryotes. *Annu Rev Biochem* **54**, 171-204 (1985).

12. Saecker, R.M. et al. Kinetic studies and structural models of the association of E. coli sigma(70) RNA polymerase with the lambdaP(R) promoter: large scale conformational changes in forming the kinetically significant intermediates. *J Mol Biol* **319**, 649-671 (2002).

13. Johnson, R.S. & Chester, R.E. Stopped-flow kinetic analysis of the interaction of Escherichia coli RNA polymerase with the bacteriophage T7 A1 promoter. *J Mol Biol* **283**, 353-370 (1998).
